# Clinical Presentation and Management of Omental Ectopic Pregnancy in a 23‐Year‐Old Patient: Integrative Case Study and Literature Review

**DOI:** 10.1155/crog/8410708

**Published:** 2026-02-23

**Authors:** Matteo Terrinoni, Michele Palisciano, Francesca Pauselli, Lorenzo Cecconi, Dario Rossetti, Gian Carlo Di Renzo

**Affiliations:** ^1^ Department of Medicine and Surgery, University of Perugia, Perugia, Umbria, Italy, unipg.it; ^2^ Department of Biomedicine and Prevention, University of Rome “Tor Vergata”, Rome, Italy, uniroma2.it; ^3^ Department of Obstetrics and Gynecology, Hospital of Città di Castello, Perugia, Italy; ^4^ Department of Obstetrics and Gynecology, Azienda Ospedaliera di Perugia, Perugia, Italy; ^5^ Department of Obstetrics and Gynecology, Hospital of Gubbio-Gualdo Tadino, Perugia, Italy; ^6^ PREIS School, International and European School of Perinatal, Neonatal and Reproductive Medicine, Florence, Italy; ^7^ Department of Obstetrics, Gynecology and Perinatology, I.M. Sechenov First State University of Moscow, Moscow, Russia

**Keywords:** ectopic pregnancy, endoscopy, gynecologic imaging, omental pregnancy

## Abstract

We describe the case of a 23‐year‐old patient presenting with suspected ectopic pregnancy, evidenced by rising beta‐hCG levels (5000 mIU/mL on March 9 and 8027 mIU/mL on March 10) and concomitant symptoms. Transvaginal ultrasound revealed a uterus with a secretory endometrium and a likely gestational pseudocavity, along with a corpuscular periuterine effusion and an abnormality adjacent to the left ovary, initially interpreted as a possible dilated fallopian tube. During laparoscopic surgery, an omental mass approximately 4 cm in diameter, suspected to represent an abdominal ectopic pregnancy, was identified. This suspicion was confirmed by intraoperative assessment, surgical consultation, and histological analysis. The lesion was excised using a minimally invasive technique, resulting in a favorable postoperative course and a progressive reduction of beta‐hCG levels (3504 mIU/mL on the first postoperative day until complete normalization on Day 10).

## 1. Introduction

Ectopic pregnancy accounts for approximately 1%–2% of all pregnancies, with the vast majority developing in the Fallopian tubes. Extratubal locations, such as the omentum, are extremely rare and present significant diagnostic and therapeutic challenges due to their atypical clinical presentation and the difficulty of preoperative identification. This case report underscores the importance of a multidisciplinary evaluation and prompt surgical intervention when an abdominal ectopic pregnancy is suspected [1–3].

## 2. Case Presentation

### 2.1. Patient History and Presentation

A 23‐year‐old woman was referred to our hospital after a routine gynecological evaluation raised the suspicion of an extrauterine pregnancy. Her past surgical history was notable for an appendectomy. Obstetric history included a preterm delivery in 2024; the neonate weighed 1400 g. The patient reported only mild pelvic symptoms and there were no signs of active, atypical vaginal bleeding on presentation.

### 2.2. Preoperative Diagnostic Work‐Up

**Figure 1:**
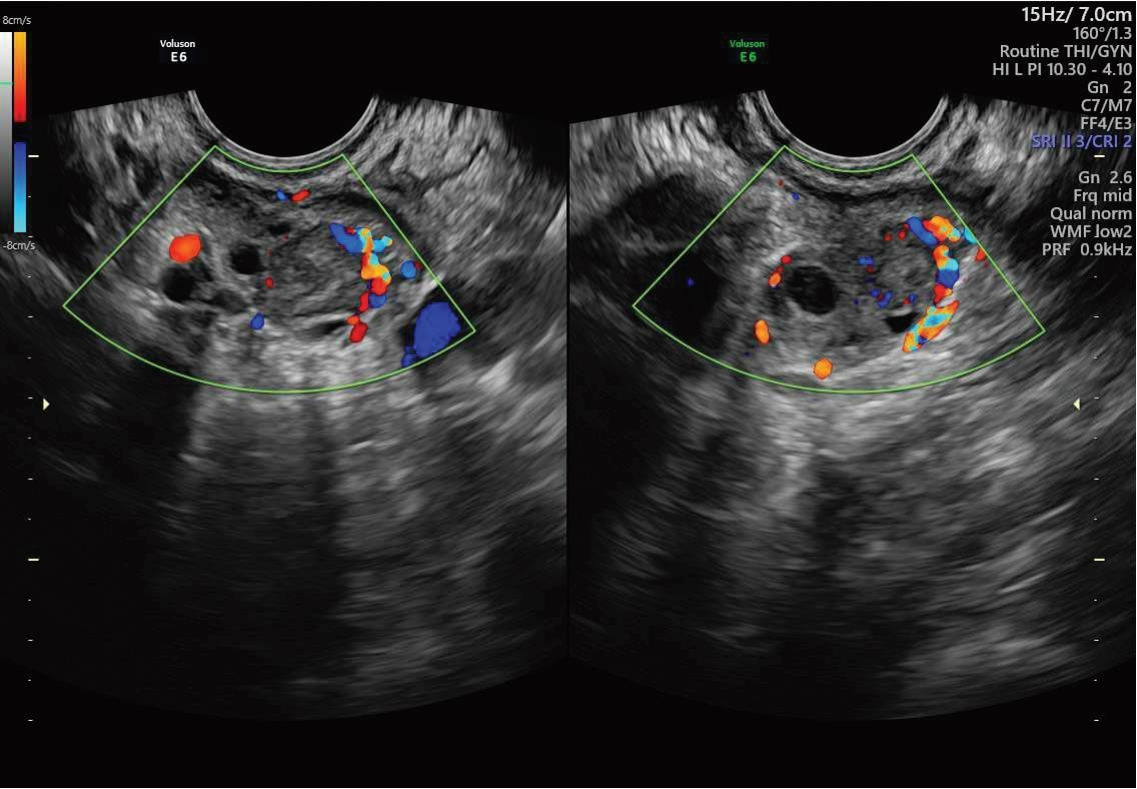
Transvaginal ultrasound at admission showing a corpus luteum on the left ovary.

**Figure 2:**
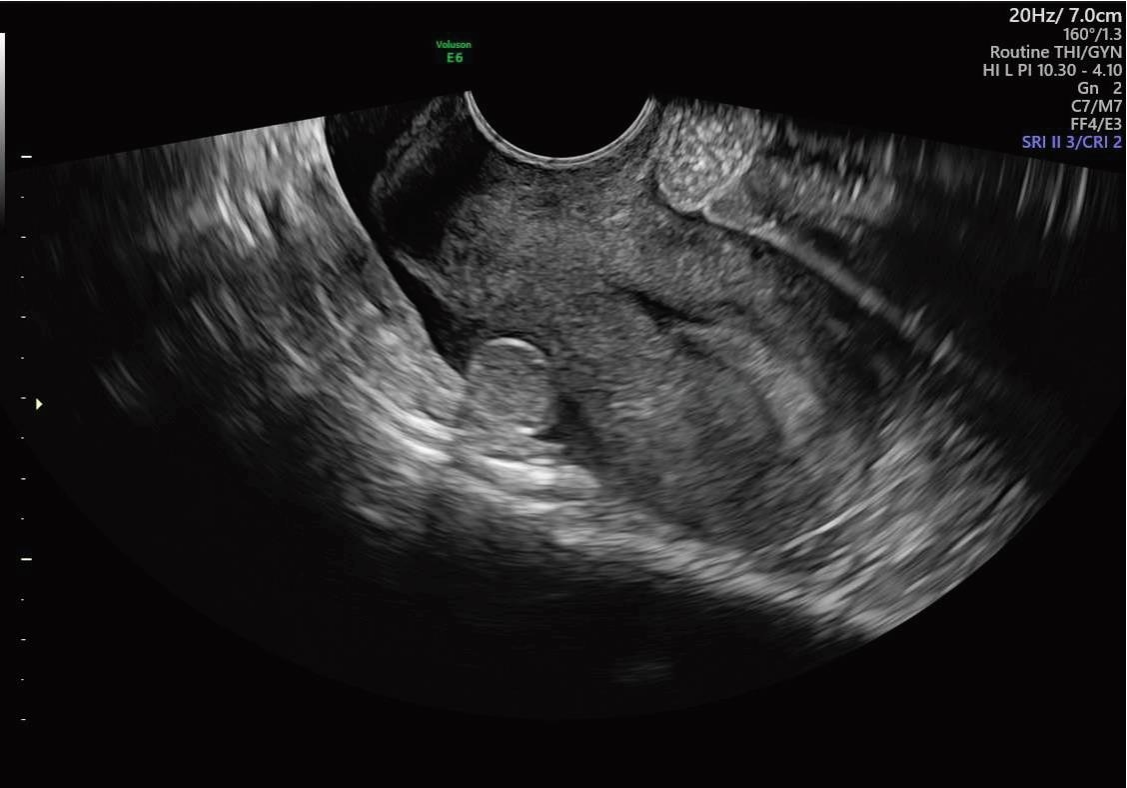
Uterus with effusion and an abnormal mass in the pouch of Douglas.

• March 9: quantitative serum *β* − hCG = 5000 mIU/mL.
• March 10 (preoperative): *β* − hCG = 8027 mIU/mL. On transvaginal ultrasound the uterus was retroverted with a secretory endometrium and a probable gestational pseudocavity. The right ovary appeared normal. The left ovary contained a corpus luteum; adjacent to it and visible in the pouch of Douglas, an abnormal mass was identified and initially interpreted as a possible dilated fallopian tube. A small periuterine particulate effusion of approximately 20 mm was also seen (see Figures 1 and 2). Given the rising *β*‐hCG and suspicious imaging, the patient was counseled and given informed consent for diagnostic laparoscopy.

### 2.3. Operative Findings and Surgical Management

**Figure 3:**
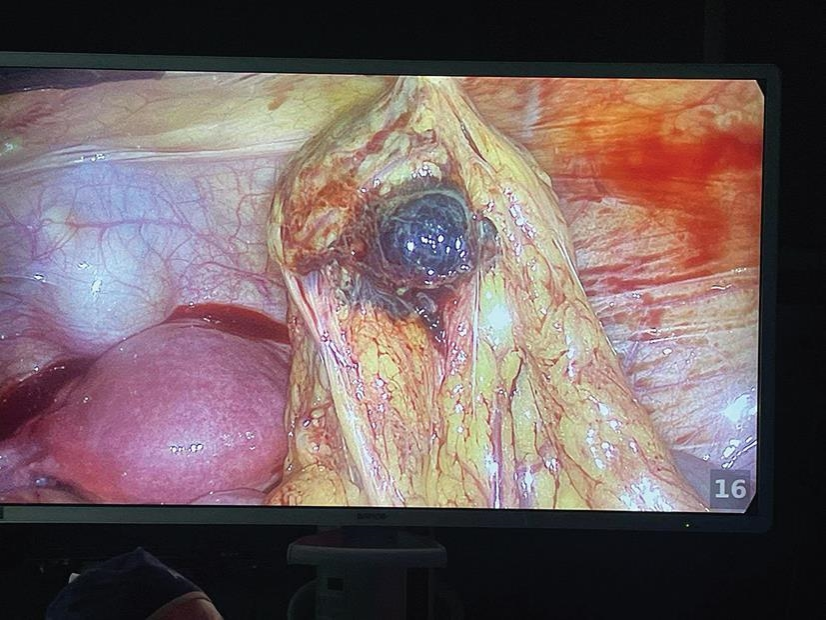
Intraoperative image depicting a 4 cm omental mass with a wine‐red appearance, located along the left inferior margin of the greater omentum.

Laparoscopy performed on 10 March revealed a modest hemoperitoneum that was immediately drained and the abdominal cavity irrigated. Inspection showed both fallopian tubes and ovaries to be intact; a corpus luteum was confirmed on the left ovary. In the pouch of Douglas, an isolated epiploic appendage ~2 cm in diameter, oval, and yellowish, was identified and sent for histological examination. Further exploration of the greater omentum disclosed an oval, firm, wine‐red lesion about 4 cm in diameter along the left inferior omental margin with surrounding edema; this omental lesion was initially suspected to represent an abdominal (omental) ectopic pregnancy (see Figure 3). After intraoperative consultation with the on‐call general surgeon, the omental margin was isolated and the lesion was excised using bipolar forceps and scissors with meticulous hemostasis.

### 2.4. Postoperative Course and Serial *β*‐hCG Monitoring

• Postoperative Day (POD) 1—March 11: *β* − hCG = 3504 mIU/mL. The immediate postoperative period was uncomplicated; the patient′s pelvic pain remained mild and there were no signs of ongoing hemorrhage or infection. On POD 2, she was discharged from the hospital.
• Serial *β*‐hCG measurements demonstrated a progressive decline; complete biochemical normalization was assumed on 20 March (*β*‐hCG within the laboratory reference range on that date). Histology of the excised specimens was submitted and confirmed the omental ectopic pregnancy.

### 2.5. Follow‐Up and Current Status

At follow‐up on 17 March, the patient was afebrile, ambulating without difficulty, and the surgical wounds were healing. She reported resolution of pelvic pain and had resumed normal activities. On 20 March, once *β*‐hCG assumed normalization, the patient was discharged from routine gynecologic follow‐up with counseling about future fertility.

### 2.6. Histology and Diagnostic Confirmation

The omental lesion and epiploic appendage were submitted for histopathology intraoperatively. The final pathology result is essential to confirm whether the omental lesion represented ectopic trophoblastic tissue (omental pregnancy), infarcted epiploic appendage, hemorrhagic fat necrosis, or another pathology.

### 2.7. Review of the Literature

Although ectopic pregnancies most commonly develop in the fallopian tubes, extratubal ectopic gestations, including those in the omentum, are extremely rare. Bouyer et al. (2003) reported that extratubal ectopic pregnancies constitute a small subset of all ectopic gestations, highlighting the inherent diagnostic challenges due to their atypical anatomical location [4]. Kirk (2012) further emphasized that the clinical manifestations of atypical ectopic pregnancies are often nonspecific, frequently resulting in ambiguous ultrasound findings that may delay diagnosis [5].

Management strategies for these rare presentations typically require a multidisciplinary approach. Wang et al. (2011) discussed the advantages of minimally invasive surgery in managing ectopic pregnancies, noting that such approaches can reduce morbidity and help preserve reproductive function even in cases of extratubal implantation [6]. Moreover, the role of medical management in ectopic pregnancies and the preoperative uncertainty in cases like omental ectopic pregnancies generally favors surgical intervention due to the elevated risk of intra‐abdominal hemorrhage [7].

Additional case reports in the literature have highlighted the variability in the clinical presentation of omental ectopic pregnancies. Some patients may be nearly asymptomatic with subtle imaging findings, whereas others may develop more pronounced symptoms that necessitate urgent intervention as currently happens for other gynecological issues [8]. The current case reinforces these findings by illustrating how an initial misinterpretation of the lesion as a dilated fallopian tube was corrected through meticulous laparoscopic exploration and surgical management.

## 3. Discussion

Omental ectopic pregnancy is an exceedingly rare condition. The clinical presentation, characterized by rising beta‐hCG levels and nonspecific ultrasound findings, necessitates a cautious and integrated approach. In this case, the initial misinterpretation of the abnormality as a possible dilated fallopian tube was rectified during laparoscopic exploration when the omental mass was clearly identified and safely removed. Minimally invasive management facilitated a rapid postoperative recovery and a significant reduction in beta‐hCG levels, thereby confirming the effective removal of ectopic tissue. Serial monitoring of beta‐hCG levels, along with histological examination, was critical in affirming therapeutic success and in excluding any residual gestational tissue [9].

## 4. Conclusion

This case highlights the importance of careful ultrasonographic evaluation and a prompt surgical approach in the management of suspected ectopic pregnancy in atypical locations. A multidisciplinary approach proved decisive in achieving an accurate diagnosis and effective surgical management, thereby minimizing the risk of complications and preserving the patient′s reproductive potential.

## Funding

No funding was received for this manuscript. Open access publishing facilitated by Universita degli Studi di Perugia, as part of the Wiley ‐ CRUI‐CARE agreement.

## Consent

All images and all information in this case report are reported under explicit informed consent of the patient: patient anonymity has been preserved in accordance with the Declaration of Helsinki.

## Conflicts of Interest

The authors declare no conflicts of interest.

## Data Availability

Data are available under reasonable request to the corresponding author.
